# Gross primary productivity analyses suggest higher ENSO-mediated impacts in lowland cacao areas compared to mountain coffee regions in Latin America

**DOI:** 10.1038/s41598-025-27292-3

**Published:** 2025-11-07

**Authors:** Andres González-González, Benjamin Quesada, Nicola Clerici, Juan Fernández-Manjarrés

**Affiliations:** 1 Université Paris-Saclay, CNRS, AgroParisTech, Laboratoire Ecologie Société Evolution, 12 route 128, 91190 Gif sur Yvette, France; 2 Earth System Science Program, School of Sciences and Engineering, Universidad del Rosario, Carrera 26 # 63b-48, Bogota D.C, 111221 Colombia; 3 School of Sciences and Engineering, Universidad del Rosario, Carrera 26 # 63b-48, Bogota D.C, 111221 Colombia

**Keywords:** ENSO, Tropical agroforestry, Species distribution models, Exposure, Sensitivity, Vulnerability, Gross primary productivity, Ecosystem ecology, Forest ecology, Agroecology

## Abstract

Agroforestry systems in Latin America and the Caribbean cover millions of hectares and are potentially vulnerable to the El Niño-Southern Oscillation (ENSO). Using a correlational approach, we examined the climatic impacts of eight El Niño and La Niña events (1992–2020) on potential coffee and cacao growing areas estimated from species distribution models. Remotely sensed canopy and subcanopy Gross Primary Productivity data (GPP) were used as a proxy for idealized two-layer agroforestry systems. Correlations with six terrestrial climate variables indicated that exposure to El Niño was ubiquitous for both potential crop zones. Hotspots where GPP decreases in both canopy and shade vegetation during El Niño and La Niña events were found mostly in Central America, Northern South America, the Western Amazon and in coastal Brazil, for both crops. Outside these zones, coastal Brazil and the southern Mata Atlântica showed a complex response to ENSO variability, particularly for potential coffee areas where increased temperatures and humidity can boost GPP. Our results raise concerns about the vulnerability of agroforestry systems, especially in potential lowland cacao areas of the Amazon, as dry El Niño events may exacerbate ongoing climate change impacts.

**Supplementary Information:**

The online version contains supplementary material available at 10.1038/s41598-025-27292-3.

## Introduction

 Agroforestry systems in the Neotropical region of Latin America and the Caribbean provide essential resources for livelihoods, conservation initiatives, and sustainable land management practices^1–3^. The two main staple crops of agroforestry systems in Latin America and the Caribbean are cacao trees in the lowlands and coffee trees in the mountainous regions. For instance, *Coffea arabica* L. is planted in subtropical broadleaf forests in the central and Northern Andes and the Talamanca cordillera in Costa Rica, while *Theobroma cacao* L. is planted in the lowlands within the tropical broadleaf forests and dry forests, similar to *Coffea canephora* L., the robusta coffee^4^. Moreover, agroforestry systems are an important strategy for climate change adaptation in the tropics^5^ and are considered as a climate-smart adaptation solution^6–8^.

However, these tropical agroforestry systems in tropical Latin America and the Caribbean are exposed to weather variability^9^, induced by teleconnections with the Pacific and Atlantic oceans. These teleconnections cause changes in the primary productivity of shade trees and of the main crops over different periods depending on the crop and region, a process that has not been quantified at the continental scale ^10^. From these two oceanic teleconnections, El Niño Southern Oscillation (ENSO) is the primary continental driver impacting vegetation activity in complex patterns^11^. ENSO variability is commonly measured using the Oceanic Niño Index (ONI), which tracks sea surface temperature anomalies in the equatorial Pacific Ocean. The ONI calculates rolling three-month averages of sea surface temperature (SST) anomalies in the Niño 3.4 region (5° N–5° S, 120° W–170° W). Anomalies exceeding ± 0.5 °C are classified as El Niño or La Niña, respectively, providing a consistent metric to analyze ENSO phases.

Contrasting dry and wet weather induced by El Niño and La Niña events can occur in the same region or during the same event in different areas of Latin America and the Caribbean. Typically, El Niño causes prolonged droughts in countries such as Colombia and Peru in South America, while La Niña causes heavy rainfall in Central America or southern Brazil^12,13^. However, coastal Ecuador and the Paraná basin in Brazil and Argentina can experience increased precipitation and not dry conditions at the onset of El Niño events, among other examples^14,15^.

The contrasting weather poses challenges to communities whose subsistence relies on multi-annual agroforestry^16^ because many fungal infections thrive during wet conditions in cacao^17^ and coffee^18^, affecting plants already weakened by previous dry weather events. Even forest trees suffer from adverse climatic conditions caused by ENSO, such as extreme temperatures, prolonged droughts, and windstorms, which collectively put them in a vulnerable state^19–21^. While the generally dry weather of El Niño can bring reduced coffee production in Mexico and Central America^22,23^, the same event brings increased production by warmer temperatures and increased precipitation during the vegetative coffee growing season in Bahia, Brazil, at the opposite side of the continent^24^. Regarding the wet and cooler climates brought by La Niña, coffee production can suffer from increased fungal diseases and reduced photosynthesis because of increased cloudiness^18^, as observed in Central America and northern South America.

Contrary to long-term studies in Africa and Asia^25,26^, existing climate extreme studies in this region are often spatially or temporally constrained, typically focusing on isolated El Niño or La Niña events. For example, some studies showed a decrease in cacao yields after the major 2015 El Niño event in Eastern Atlantic Brazil^27^ or increased coffee yields in Southern Brazil in the early stages of recent El Niño events^24^. Also, increased tree mortality of certain agroforestry species after ENSO events has been documented^28^.

While assessing exposure to climatic teleconnections for agroforestry areas is possible with current climate databases, determining the sensitivity of the photosynthetic levels at large spatial scales for agroforestry systems poses additional challenges. The critical characteristic of a canopy structure consisting of at least one crop of interest with a tree canopy distinguishes agroforestry systems from tree monocultures. In Latin America and the Caribbean, for example, canopy species include, but are not limited to, many leguminous shade trees (*Inga* spp., *Erythrina* spp.)^29^. The photosynthetic activity and the associated water use of the shade trees significantly influence the productivity of the lower vegetation layer where the crop of interest is grown, potentially competing for water with the shade trees^30^. These signals of photosynthetic activity, which can be captured by remote sensing^31^, can indicate the sensitivity of agroforestry systems to environmental stressors, providing valuable insights into their resilience and adaptability to new climates^32^.

Most remotely sensed vegetation studies in the tropics and Latin America and the Caribbean addressing the teleconnections with ENSO have focused mainly on assessing the impact on the gross productivity of the vegetation as a whole. For instance, indices such as NDVI have effectively captured the influence of atmospheric teleconnections between ENSO and crop production, forest ecosystems, and natural vegetation^33–35^. Zhang et al. (2019)^36^ analyzed ENSO and Gross Primary Production (GPP), identifying several areas in Latin America and the Caribbean where ENSO negatively impacts GPP, but did not specify which ecosystems or crops would be more affected by climate fluctuations. Therefore, there remains a critical need to examine productivity dynamics at the canopy and within the subcanopy layers when studying agroforestry systems.

The big-leaf and two-leaf Light Use Efficiency (LUE) GPP models are essential tools for analyzing productivity in agroforestry systems. Cui et al. (2024)^37^ compared six big-leaf LUE-GPP models and one two-leaf LUE-GPP model with eddy covariance-Light Use Efficiency (EC-LUE) models in a humid subtropical agroecosystem region. The two-leaf model outperformed the big-leaf models by simulating radiation transmission through the canopy and accounting for differences in photosynthetic capacity between sunlit and shaded leaves. Despite its advantages, some limitations remain, including occasional under- or overestimation of GPP under varying weather conditions.

One approach to addressing multi-strata vegetation productivity was presented recently by Bi and collaborators^38^, who generated a high spatial resolution dataset from an updated two-leaf light use efficiency (TL-LUE) model (reviewed by Cui et al. (2024)). In their approach, Bi and colleagues distinguished between the gross primary production of sunlit and shaded leaves (hereafter GPPsun and GPPshade). Conceptually, sunlit leaves absorb both direct and diffuse radiation simultaneously, making them more susceptible to light saturation, especially at high radiation intensities. Conversely, shaded leaves absorb mainly diffuse radiation. The level of absorbed radiation in shaded leaves usually falls between the light compensation and saturation points^39,40^. All else being equal, we can assume that a proxy for the productivity of agroforestry systems would be provided by the GPP of sunlit and shaded leaves, allowing us to detect patterns at large continental scales of canopy versus subcanopy vegetation responses to climate variability.

Finally, another shortcoming of continental-scale agroforestry studies is the lack of comprehensive maps of the main agroforestry systems. According to Somarriba et al. (2012)^1^, agroforestry is estimated to cover between 200 and 360 million hectares in Latin America and the Caribbean, with 14–26 million hectares in Central America and 88–315 million hectares in South America, but accurate estimates of their current extent remain unknown. In this regard, species distribution models (SDM) can provide robust large-scale estimates for the potential distribution of agroforestry systems for species of coffee and cacao under current conditions and climate change^41^.

To the best of our knowledge, no previous study has used remote sensing data to examine the responses of agroforestry areas at a continental level and during various episodes of EL Niño/ La Niña. In this study, we examined the exposure and sensitivity of potential and current agroforestry areas in the tropical region of Latin America and the Caribbean (i.e., 23.5º *N* − 23.5º S) to the climatic fluctuations of the El Niño-Southern Oscillation (ENSO) over a 29-year period (1992–2020). In our approach, we followed the notions in which “exposure” is defined as the nature and degree to which a system experiences environmental stress and “sensitivity” as the degree to which a system responds to perturbations^42–44^. Within this tropical area, we focused on the potential distribution of coffee and cacao under current climates as examples of middle-elevation mountain and lowland agroforestry systems, respectively. We asked: *i*) What is the current climatic exposure of potential coffee and cacao growing areas to ENSO teleconnections across the tropical region in Latin America and the Caribbean? *ii*) What can multi-strata Gross Primary Production (GPP) estimates tell us about the sensitivity of potential coffee and cacao growing areas to ENSO variability? And, *iii*) What is the temporal relationship between the ENSO fluctuations and the GPP sensitivity in these potential coffee and cacao areas?

## Results

### Species distribution models for *Coffea arabica* and *Theobroma cacao*

**Fig. 1:**
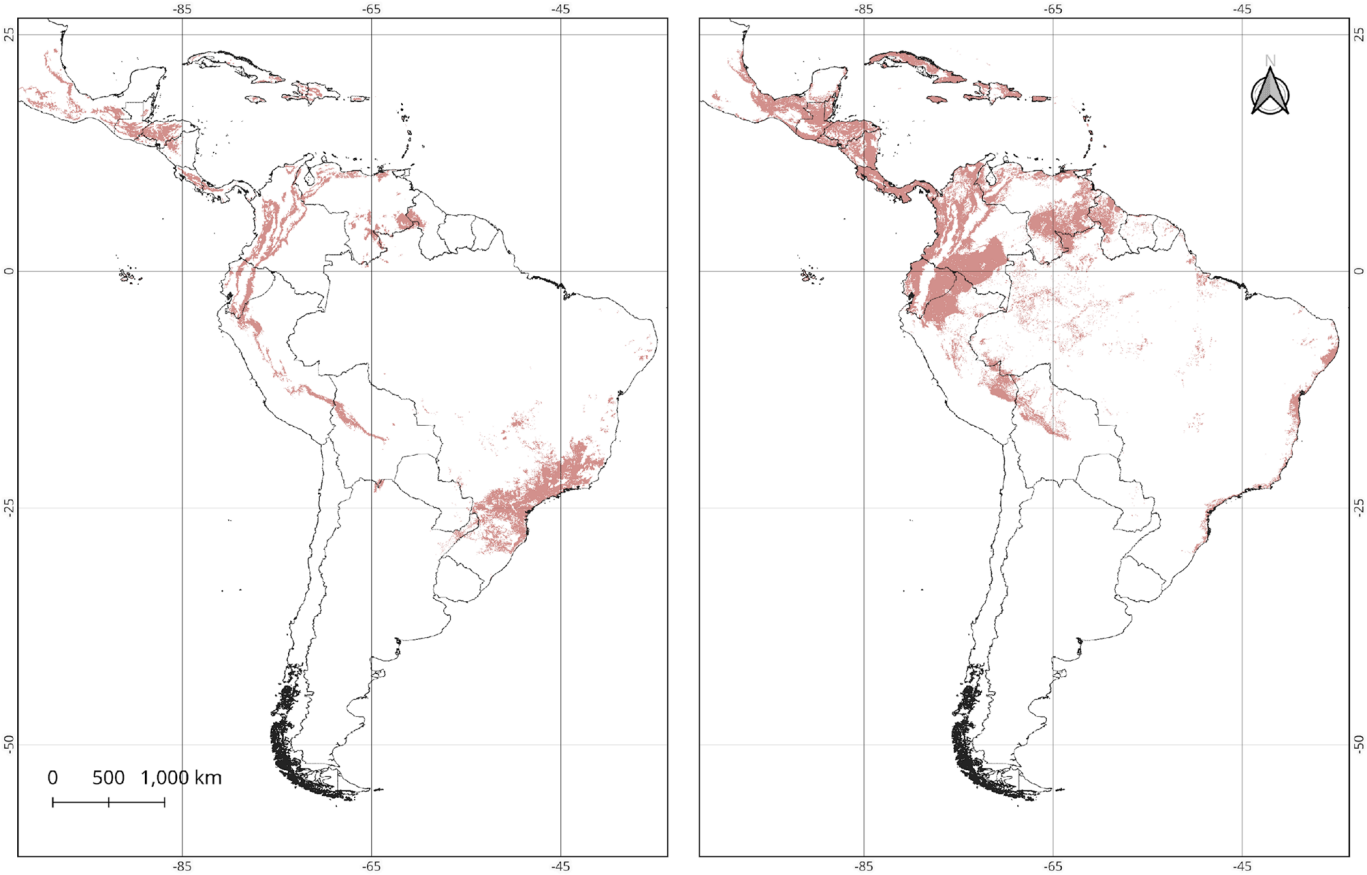
Maximum entropy distribution models for *Coffea arabica* (left panel) and *Theobroma cacao* (right panel) based on GBIF datasets and using six environmental variables plus soil type and biome classification In practice, the exact location of these crops is more scattered because they depend on microclimates and soil conditions that are not captured by the model at this large scale. The continuous MaxEnt output was transformed into a binary distribution using the Test Specificity Training threshold, which provides a conservative estimate of the true distribution. Map created using QGIS v3.34.2-Prizren (QGIS Development Team, 2024; https://qgis.org).

Figure 1 depicts the potential zones for coffee and cacao based on the MaxEnt approach^45^. The cross-validation of 10 independent runs for each species showed that the Biome classification layer and minimal temperature, followed closely by maximum temperatures contributed more to the distribution model of *C. arabica*. In contrast, minimum temperature, soil classification and maximum temperature were the variables that contributed more to the distribution model of *T. cacao*. Overall, the predictive value of the SDM model was better for coffee than cacao. The average test data for the area under the curve (AUC), a measure of discriminative power between the presence and absence of the models, was 0.893 and 0.855 for coffee and cacao, respectively. The AUC values of the models are similar to other studies conducted with high-resolution record data in smaller areas^46,47^. Compared to intensively studied areas of coffee and cacao production in Brazil (see online methods for references), our simulations produced a proportion of true positives of 87% and false positives of 13% for coffee. Likewise, the proportion of true positives was 79%, and the false positives was 21% for cacao (see Figure S7 in the Supplementary Information).

### Atmospheric teleconnections as a proxy of climatic exposure of agroforestry systems

**Fig. 2:**
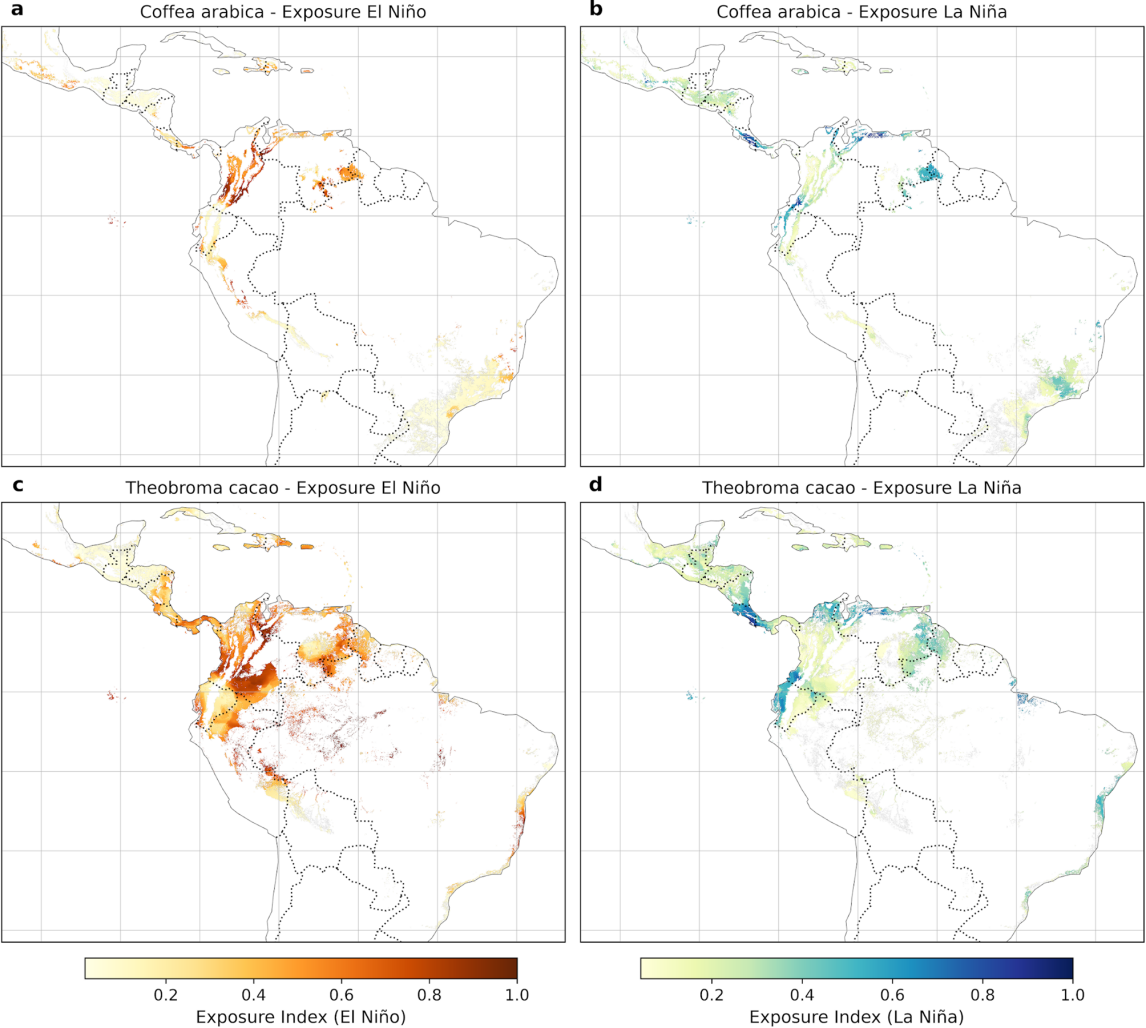
Spatial distribution of the climatic exposure index calculated using the Entropy Weight Method (EWM) for *Coffea arabica* (**a**, **b**) and *Theobroma cacao* (**c**, **d**) predicted suitability areas under El Niño (**a**, **c**) and La Niña (**b**, **d**) conditions. The exposure index ranges from 0 to 1, with warm colors (yellow to brown) indicating areas highly exposed to drought-related stress during El Niño and cool colors (green to blue) representing areas exposed to wetness-related stress during La Niña. Dashed lines represent country borders. See Figures S1 and S2 for correlation results between ONI and each of the six variables. Maps generated in Python v3.11 using Matplotlib v3.10.3 (https://matplotlib.org*)* and Cartopy v0.24.1 (https://scitools.org.uk/cartopy*).*

While climatic exposure to El Niño conditions is ubiquitous in the potential coffee and cacao zones predicted by our SDMs, climatic exposure to La Niña conditions is more localized to lowland areas (Fig. 2). These patterns also illustrate that El Niño primarily leads to drought-related exposure, and La Niña results in wetness-related exposure during four El Niño events (1991–92, 1997–98, 2009–10, and 2015–16) and four La Niña events (1998–99, 1999–00, 2007–08, and 2010–11) (NOAA 2023a). These correlations of the Oceanic Niño Index ONI time series with the terrestrial climatic variables from 1992 to 2020 suggest that for most areas, land temperatures increased as Pacific temperatures increased, especially minimum temperatures and that precipitation decreased for both coffee and cacao potential areas (Figure S1a–c). The selected derived variables of agroecological importance (Palmer Drought Severity Index, soil moisture) closely follow precipitation patterns (Figure S1d,e), except for vapor pressure deficit, which shows opposite patterns to precipitation (Figure S1d).

#### El Niño exposure of *Coffea arabica* and *Theobroma cacao*

Under El Niño conditions, climatic exposure for the *Coffea arabica* predicted suitability area is widespread. Entropy values were high and ranged from 0.631 for vapor pressure deficit (VPD) to 0.745 for minimum temperature (Tmin). Entropy values close to one indicate a spatially homogeneous effect of the variables. Overall, VPD received the greatest weight (0.1940), followed by precipitation (0.1754), soil moisture (0.1728), PDSI (0.1709), Tmax (0.1529), and Tmin (0.1340) (Table 1). Geographically, el Niño-induced climatic exposure of coffee areas was particularly strong in the Pacific slopes of the Central American mountain ranges, Haiti/Dominican Republic, the northern Andes, and the southern coastal Mata Atlântica of Brazil (Fig. 2a).

**Table 1:** Entropy and weights for the exposure multivariable index for coffee and Cacao estimated for ENSO-El Niño and ENSO-La Niña between 1992 and 2020.

Variable	C. Arabica potential zones				T. cacao potential zones			
	El Niño		La Niña		El Niño		La Niña	
	Entropy	Weight	Entropy	Weight	Entropy	Weight	Entropy	Weight
Tmax	0.7093	0.1529	0.7109	0.1441	0.7535	0.1698	0.7441	0.1516
Tmin	0.7453	0.1340	0.6809	0.1591	0.7893	0.1452	0.7281	0.1610
VPD	0.6312	**0.1940**	0.6453	**0.1768**	0.7334	**0.1837**	0.6822	**0.1882**
pr	0.6666	0.1754	0.6513	0.1738	0.7526	0.1704	0.7146	0.1690
soil	0.6714	0.1728	0.6458	0.1765	0.7527	0.1704	0.7124	0.1703
pdsi	0.6751	0.1709	0.6597	0.1696	0.767	0.1605	0.7301	0.1599

The highest weights contributing to the vulnerability index are presented in bold.

For the *Theobroma cacao* predicted suitability area, entropy values were higher than for coffee and ranged from 0.7334 (VPD) to 0.7893 (Tmin). Weights for the variables in decreasing order were VPD (0.1837), precipitation (0.1704), soil moisture (0.1704), Tmax (0.1698), PDSI (0.1605) and Tmin (0.1452) (Table 1). Similar to the predicted suitability range for coffee, VPD appeared to be a high contributor to the EWI during El Niño for *T. cacao*. The climatic exposure of potential cacao areas (Fig. 2c) forms an almost continuous stretch of lowlands where most variables studied show significant correlations between the ONI index and terrestrial meteorological variables (Figure S1a–f). Almost all of Nicaragua, Costa Rica, Panama, and Colombia were affected during the studied EL Niño events, as well as the eastern and western regions of Haiti and the Dominican Republic, respectively, the eastern Peruvian Amazon lowlands and the coast of Brazil (Espirito Santo and Bahia states). Nevertheless, El Niño events during the study period were also correlated with increased precipitation over much of Guatemala, the dry forests of coastal Ecuador, and the Paraná basin in Brazil, while causing the well-known droughts elsewhere for both coffee and cacao (Figure S1c).

#### La Niña exposure of *Coffea arabica* and *Theobroma cacao*

During La Niña conditions, *C. arabica* predicted suitability areas displayed entropy values from 0.6453 (VPD) to 0.7109 (Tmax). Similar to El Niño, VPD exhibited the highest weight (0.1768), followed closely by soil moisture (0.1765), precipitation (0.1738), PDSI (0.1696), Tmin (0.1591), and Tmax (0.1441) (Table 1). These results indicate that VPD also remains a critical driver during La Niña events, although the relatively high weights for soil moisture and precipitation reflect the increased importance of wetness-related stressors. The La Niña-induced climatic exposure for coffee areas overlaps with many areas affected by El Niño. These double exposed areas are mostly localized between Costa Rica and Panamá for Central America, northern Colombia (Sierra Nevada de Santa Marta), the Andes and the northern coast of Venezuela, the Andean zone between Colombia and Ecuador, and the Guiana Shield, a region that can be considered a hotspot for continuous ENSO impacts. In contrast, the southern part of the Mata Atlântica, which can also be considered a hotspot, has an opposite direction of the changes in the meteorological variables (Fig. 2b, Figure S2a–f).

For the predicted *T. cacao* suitability areas, entropy values ranged from 0.6822 (VPD) to 0.7441 (Tmax). The weights in decreasing order were VPD (0.1882), soil moisture (0.1703), precipitation (0.1690), Tmin (0.1610), Tmax (0.1516), and PDSI (0.1599) (Table 1). Similar to Coffea, VPD maintained its dominance, but the slightly higher weights for precipitation and soil moisture reflect their significance under wetter conditions. These lowland areas exposed to La Niña were found from Guatemala to Costa Rica, the coast of Ecuador, northern South America, the northern part of the Guiana Shield and the areas of the Amazon River delta (Fig. 2d). With a few exceptions, areas with teleconnections to El Niño are also prone to teleconnections to La Niña events.

When we compared the climatic exposure index values for the 1961–1990 and 1991–2020 periods, our analyses show a strengthening of the El Niño teleconnections and a weakening of La Niña, especially in northern South America (Figure S3), a result that can only be confirmed with future observations.

### Gross primary productivity as a proxy of sensitivity for agroforestry systems

**Fig. 3:**
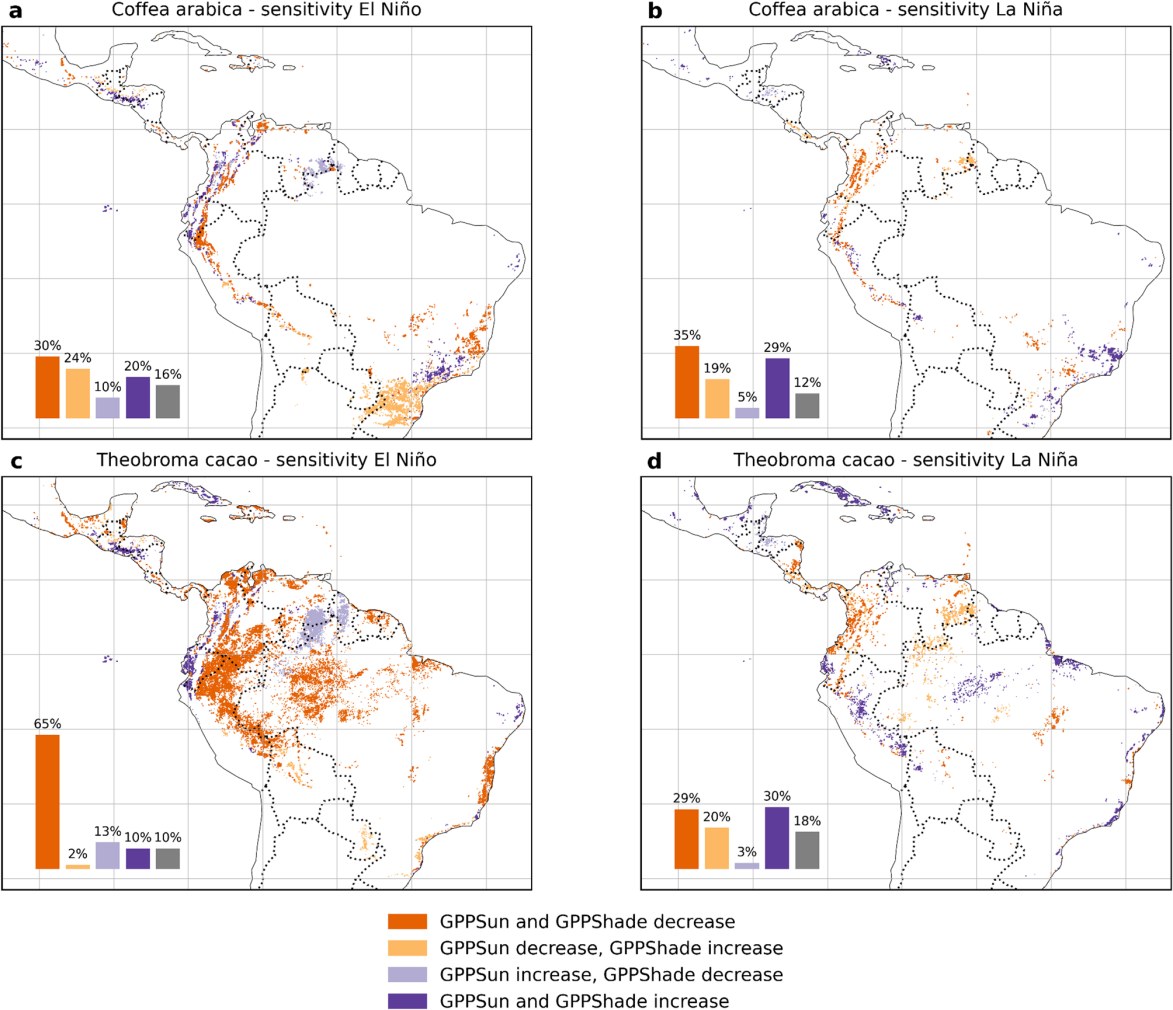
Behavior of the Pearson correlation coefficients (*p* < 0.05) from 1992 to 2020 between ONI and GPP for potential coffee (**a**, **b**) and cacao (**c**,** d**) zones for sunlit and shaded leaves. Correlations were converted to binary values to indicate a positive or negative effect on GPP on sunlit and shaded leaves. The direction of the response of sunlit or shaded vegetation is color-coded according to the legend. Bars indicate the percentage of pixels containing each of the four categories of GPP responses. Areas do not add up to 100% as only non-deforested pixels were chosen for the analyses. Dashed lines represent national boundaries. Please refer to Figures S4–S5 for the original correlation values. Maps generated in Python v3.11 using Matplotlib v3.10.3 (https://matplotlib.org*)* and Cartopy v0.24.1 (https://scitools.org.uk/cartopy*).*

The correlations of Gross Primary Production for leaves exposed to direct sunlight (GPPSun) and to leaves exposed to indirect sunlight (GPPShade) can provide insights about the direction of the vegetation responses. El Niño had negative effects on both GPPSun and GPPShade productivity almost everywhere for the potential zones of both crops (dark orange pixels in Fig. 3a and c), but especially for cacao with 65% of its potential areas affected (Fig. 3c). On the other hand, positive effects of El Niño were observed in the dry forest of the Ecuadorian coast and in the very humid forests of the Pacific slope of the Andes in Colombia and some parts of Central America representing 20% and 10% for coffee and cacao areas, respectively (purple pixels in Fig. 3a and c). Areas where the canopy, but not the subcanopy, was less productive during an El Niño event appeared in the potential coffee distribution towards southern Brazil in the Paraná basin (light orange pixels in Fig. 3). The opposite case, where the subcanopy has reduced productivity but not the canopy, appeared concentrated in the Guyana shield for both crops (light purple pixels in Fig. 3a and b).

With respect to La Niña, our results indicate increased productivity for potential cacao areas in the Caribbean, the eastern slope of the Andes in Peru, and the Amazon lowlands (purple pixels in Fig. 3d), while decreasing productivity in the inter-Andean valleys and the Pacific coast of Colombia, as well as parts of the Central American lowlands (dark orange pixels in Fig. 3d). In the case of potential coffee areas, La Niña reduced productivity almost everywhere except for parts of coastal and southeastern Brazil (dark orange pixels in Fig. 3b). Areas where the canopy, but not the subcanopy, showed lower productivity during La Niña events occur in the northern Amazon Basin and the Guyana Shield for both crops (light orange pixels in Fig. 3b and d).

### Timing ENSO impacts on gross primary productivity

To examine both the timing and geographic extent of productivity responses to ENSO variability, we performed time lag analyses of the ONI-GPP correlations covering three months before and three months after the ONI peak (Figures S4 and S5). We specifically focused on the months for which changes in the median response of GPP could be observed.

**Fig. 4:**
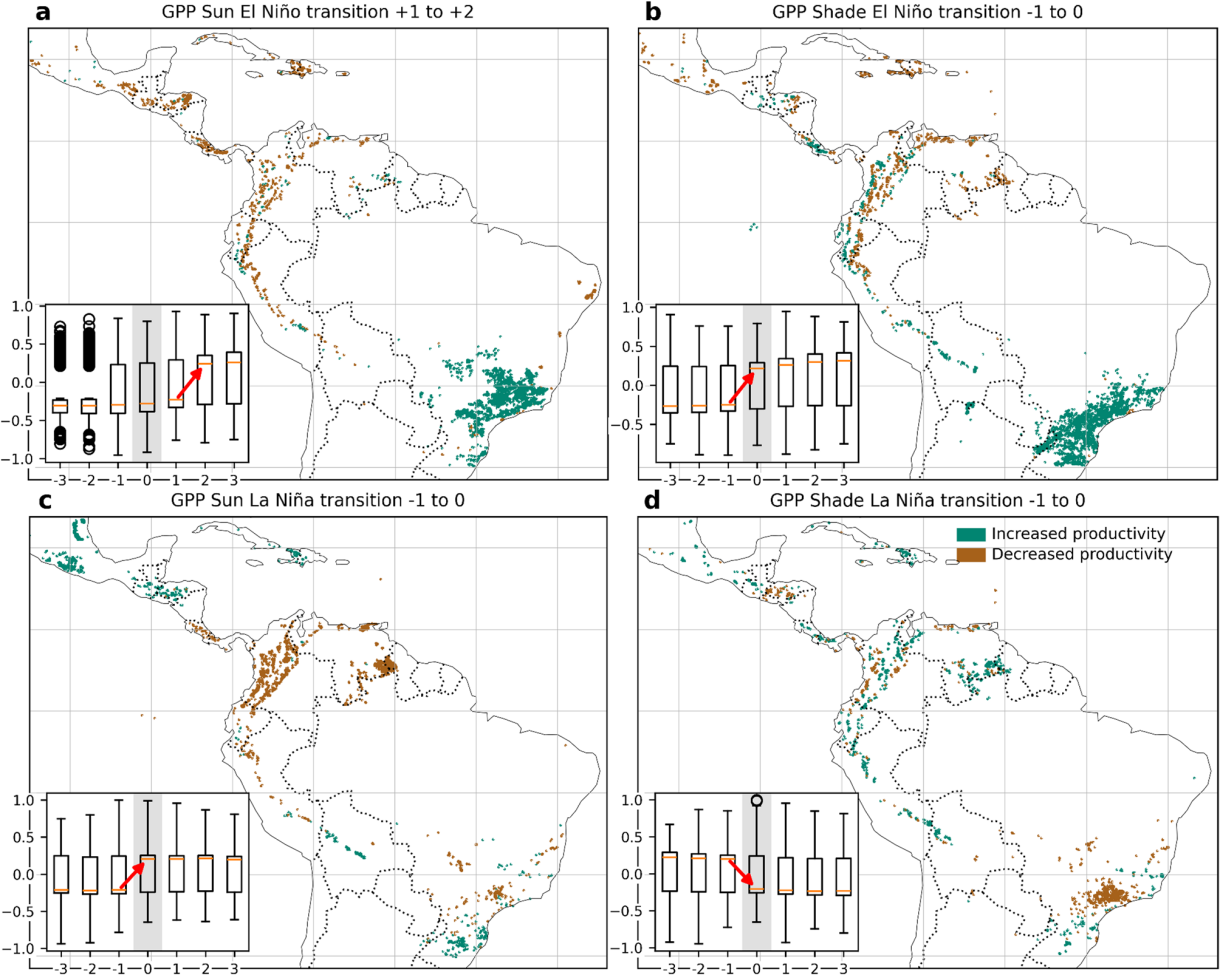
Spatial and temporal patterns of changes in gross primary productivity (GPP) response across time lags during ENSO events in potential coffee-growing zones. Panels (**a**, **b**) represent El Niño conditions for Sun and Shade layers, respectively; panels (**c**, **d**) show La Niña conditions. Colored areas indicate zones where correlations between the Oceanic Niño Index and GPP shifted from non-significant to significant across time lags. Green areas indicate increased productivity (i.e., positive correlations during El Niño or negative correlations during La Niña), and brown areas indicate decreased productivity (i.e., negative correlations during El Niño or positive correlations during La Niña). Each inset boxplot shows the distribution of pixel-wise correlation coefficients (*r*) for lag months − 3 to + 3; lag 0 highlighted in light grey corresponds to the non-lagged ONI-GPP correlation used in Fig. 3. The four panels show transitions at different moments chosen according to the two time lags in which the median response exhibited a clear change in direction: months + 1 to + 2 (**a**), −1 to 0 (**b**,** c** and **d**), marked with red arrows in each boxplot. Maps generated in Python v3.11 using Matplotlib v3.10.3 (https://matplotlib.org*)* and Cartopy v0.24.1 (https://scitools.org.uk/cartopy*).*

For potential coffee zones the transitions from increased to decreased productivity and vice versa were highly geographically concentrated and did not occur at the same time (Fig. 4 and Figures S4 to S5). During El Niño events, coffee zones experienced an initial increase in productivity after the start of the event (lags 1 to 2) in the canopy (Fig. 4a) but before the start of the event (lags − 1 to 0) in the subcanopy (Fig. 4b) in Southeastern Brazil and in the Eastern Andes of Peru and Bolivia. In contrast, the Andes of Colombia and Venezuela, Central America and the Caribbean experienced decreased productivity in potential coffee zones, except for patchy places.

On the other hand, during La Niña events, potential coffee canopy vegetation layers experienced a sustained decline in productivity from Nicaragua, the Caribbean, northern South America, and parts of southeastern Brazil compared to the previous month of a full-blown event (lags − 1 to 0, Fig. 4c). However, the subcanopy layer increased productivity also within one month before full-blown La Niña events for coffee potential zones in the Andean countries and many parts of Central America and the Caribbean, while clearly decreasing in southeastern Brazil, an effect remaining permanent for three months (Fig. 4d).

**Fig. 5:**
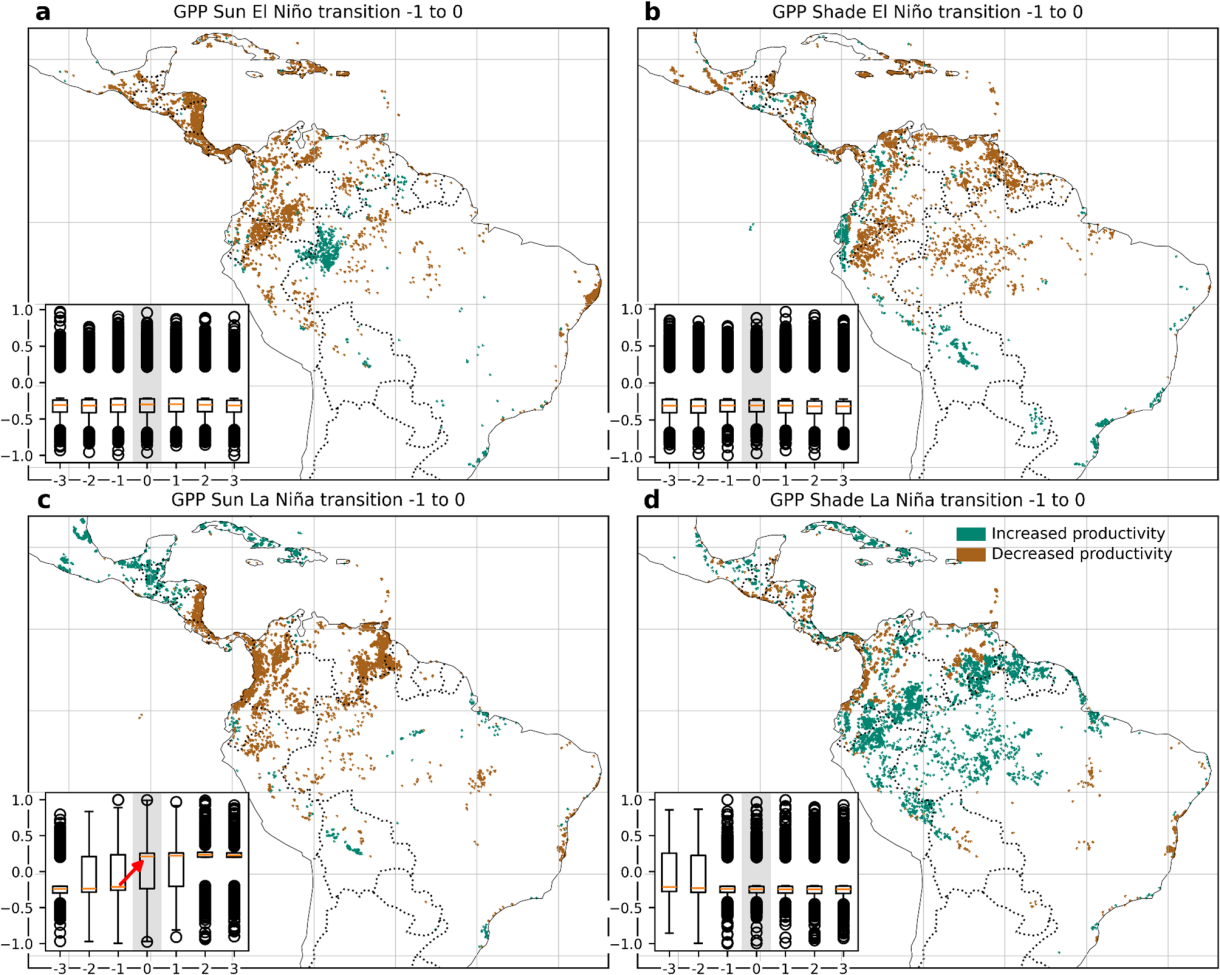
Spatial and temporal patterns of changes in gross primary productivity (GPP) response across time lags during ENSO events in potential cacao-growing zones. Panels (**a**, **b**) show GPP responses under El Niño conditions for Sun and Shade layers, respectively, while panels (**c**, **d**) show responses under La Niña conditions for Sun and Shade layers. Colored areas indicate zones where correlations between the Oceanic Niño Index (ONI) and GPP shifted from non-significant to significant across time lags. Green areas indicate increased productivity (i.e., positive correlations during El Niño or negative correlations during La Niña), and brown areas indicate decreased productivity (i.e., negative correlations during El Niño or positive correlations during La Niña). Each inset boxplot shows the distribution of pixel-wise correlation coefficients (*r*) for lag months − 3 to + 3; lag 0 highlighted in light grey corresponds to the non-lagged ONI-GPP correlation used for Fig. 3. Only one clear transition in the magnitude of the median was observed during La Niña for the Sun layer (red arrow in panel **c**). For consistency, the same − 1 to 0 transitions were depicted for panels **a**, **b** and **d**. Maps generated in Python v3.11 using Matplotlib v3.10.3 (https://matplotlib.org*)* and Cartopy v0.24.1 (https://scitools.org.uk/cartopy*).*

Potential cacao zones remained under constant stress before, during and after El Niño events, although with a large variability probably related to microclimate and local conditions (Fig. 5). Because no clear direction of change was observed for the cacao areas, except GPP Sun la Niña transition (Fig. 5c), we used the same time lag transition of −1 to 0 months in all the analyses. During El Niño phases, almost all of the lower tropical lands in Latin America and the Caribbean are experiencing decreased productivity in the canopy at the onset of El Niño, except for one area at the core of the Amazon (Fig. 5a). GPP shade on parts of Central America, the Pacific coast, Eastern Andes of Peru and Bolivia and the Atlantic coast in Brazil appeared with increased values (Fig. 5b) while all the lowlands north and east of the Andes appeared with decreased GPP values suggesting a widespread El Niño negative impact since the start of the event.

A clear temporal transition effect for cacao potential areas and La Niña was observed for the canopy layer showing a decrease in GPP for almost all of South America and Central America but that induces GPP increases for Mexico and the Caribbean before the full-blown event (Fig. 5c). However, the subcanopy of potential zones for cacao experienced an increase in productivity due to La Niña in the Amazon basin, Guiana shield and in Guatemala and the Caribbean compared to the previous month of the event, a phenomenon observed in almost all of the study area except for Central America and the Pacific coast of Colombia (Fig. 5d).

## Discussion

Overall, our results confirm observations from the literature regarding positive effects of El Niño in the coffee and cacao growing areas of coastal southern Brazil, including the Paraná region, but draw a less enthusiastic view for other parts of Latin America and the Caribbean, including the northern Andes and Central America. Of more concern is the widespread vulnerability of potential cacao agroforestry areas if they continue to develop in the Amazon basin, as GGP appears permanently compromised during the studied years. A more optimistic finding, however, is that La Niña events can counter GPP reductions in the Amazon region, a finding that merits careful attention for future research. Impacts on the Caribbean appear to be moderate, but strong enough to warrant serious consideration.

Our results revealed high and continuous climatic vulnerability zones stretching from Central America to northern South America comprising moist lowland broadleaf forests where cacao and other species with similar ecology are potentially grown. For the period analyzed (1992–2020), humid tropical lowlands are negatively affected by El Niño both at the canopy and subcanopy strata (65% of the areas in Fig. 3c), especially in the Amazon basin, making this zone of highest concern for the development of agroforestry. In addition, la Niña’s possible light-limiting effects on photosynthesis^48^ were also evident, especially for potential cacao zones in the inter-Andean valleys and the Pacific coast of Colombia, as well as parts of the Central America lowlands down to Belize in the Caribbean basin. For potential coffee areas, La Niña reduces productivity almost everywhere except for parts of coastal and southern Eastern Brazil. Nevertheless, teleconnections with south Eastern and coastal Brazil appeared not as strong as those from Central America and northern South America, but are strong enough to produce favorable conditions for increased coffee production during El Niño episodes, as we discuss below.

We observed consistent climatic exposure patterns between ONI-precipitation, ONI-soil moisture, and ONI-Drought SI correlations, suggesting a complex and strong interplay between ENSO dynamics and variables important for agroforestry. In particular, the water vapor pressure deficit (VPD), which has been used to identify thresholds affecting coffee production^49^, also showed high levels of correlation (teleconnections) with ONI (see Figs. S1 and S2). Moreover, our results showed that VPD is the largest contributor to the exposure index (Table 1; Fig. 1). In this regard, the use of agroforestry systems to buffer regional variation of VPD is coherent with a climate change adaptation strategy of maintaining high water air saturation by the use of shade trees (see for example, Avila-Lovera et al.^50^). Also, VPD can serve for local monitoring processes as it is easy to calculate from local meteorological data.

In areas where the GPP sun is negatively affected, agroforestry systems may already be at risk if the trees used for shade are already experiencing water stress. Our analysis showed that both the Andes region and South Eastern Brazil both exhibited reduced GPP for the canopy level during El Niño events (Fig. 3a). La Niña events appeared to produce the same effect on canopy trees in potential coffee and cacao areas mostly in the Andean region and Central America (Fig. 3b and d). In fact, these areas may represent cases where the target crops may already be under stress if they are in competition with their shade trees as has been observed in Africa for cacao systems^51^. Again, southeastern Brazil was not impacted during the study period.

Certain regions in our analyses appeared to be favored by El Niño or La Niña. These regions include the Andean coffee region between Venezuela and Bolivia, the arid Pacific coast of Mexico, and the grasslands and savannas of southeastern Brazil and the Paraná basin. In fact, Almeida Silva et al.^24^ observed positive effects of El Niño throughout Brazil during the period 2002–2017, when warmer temperatures promote vegetative growth^52^. Overall, the largest positive effect in our data was observed for potential cacao areas during the full-fledged La Niña events on the Amazon region, parts of Central America and the Caribbean, where the wet conditions clearly bring increased GPP for the sub-canopy layer of vegetation (Fig. 5d).

Resilience to the dry conditions of El Niño may be highly dependent on deforestation rates across the landscape where agroforestry is practiced. Evidence has been accumulating for several years on how deforestation thresholds can locally alter climate and vegetation in the Amazon^53^. Typically, agroforestry systems target tree shade canopy cover of no more than 60%^54^, and several studies have found forest sensitivity even above a 30% deforestation threshold (Flores et al. 2024^55^ and references therein). It is unknown what percentage of tree cover will actually be required if the microclimates in the Amazon and surrounding seasonal forest ecosystems continue to dry out due to large-scale deforestation (for example, Machado et al.^56^). In this sense, potential agroforestry systems could be implemented with a diversified shade cover to maximize the presence of resilient species, as in the Cabruca cacao agroforestry in the Atlantic Forest of Brazil^57^.

It is important to consider that our approach may underestimate the effects of ENSO on real agroforestry systems. The GPP data that we used were designed to separate leaves under open sun and all the other layers as a single unit. However, agroforestry systems may have different tree densities and more importantly, more than two tree strata. Recent reviews of the effects of different agroforestry systems have shown that the interplay between the density of cacao trees and the spatial arrangement of shade trees revealed a complex response (see Mattalia et al.^58^ and references therein). Our results are therefore, likely to be more appropriate for very complex agroforestry systems such as the Cabrucas cited above^59^ or the type of complex Amazonian cacao systems in Colombia, where at least 127 species of native trees are kept for shade^60^.

In sum, there is a spatially heterogeneous sensitivity of GPP to ENSO teleconnections, rather than a single large effect with negative and positive outcomes. Overall, Southeastern Brazil appeared moderately or even positively affected by ENSO fluctuations. However, our results raise more concern for the Amazon region, the Andes, and parts of Central America and the Caribbean, where warm and dry conditions may offset any initial positive effects of ENSO events. Our analyses also showed scattered areas with positive GPP effects during ENSO events, a result that might reflect micro-habitat advantages. Therefore, strategies and policies for dealing with ENSO uncertainty should be site-specific. Overall, the potential lowland cacao areas appear to be strongly affected by El Niño events, calling for careful implementation of agroforestry systems that can maintain the most resilient native species to avoid the dieback of shade trees. Considering that the time lags are very short because the teleconnection effect is almost immediate and the GPP effects last for at least three months, it is important that agricultural managers and authorities continuously monitor the early warming provided by NOAA and other institutions before the ENSO events fully develop.

The opposing phases of ENSO generate contrasting weather conditions that demand divergent management strategies: during El Niño, shade may be essential to mitigate heat and water stress in countries like Colombia, Venezuela or Costa Rica, whereas under La Niña, full sun exposure could help control humidity-related diseases. This tension of opposing management strategies for ENSO variability may suggest that in these countries, a type of sparse agroforestry may become popular in contrast to close shade, at least for coffee plantations in mountainous areas. For lowland areas where cacao is typically grown, like the Amazon basin, the use of drought-resistant varieties seems essential even for dense agroforestry systems.

## Online methods

### Simulating potential zones for coffee and Cacao agroforestry

The main challenge in studying climate impacts on crops at the continental scale is the lack of comprehensive and up-to-date land use maps for major crops in Latin America and the Caribbean. In addition, crops such as coffee and cacao are often grown in agroforestry systems under shade trees, which are typically assimilated to mixed agricultural areas in land use maps, thus underestimating this type of multi-layered agricultural system. Previous studies have proposed distribution models for coffee or cacao from farm information^61^, but complete datasets with precise locations are not yet publicly available. Therefore, we applied a species distribution model(SDM) approach using observations in the GBIF database for *Coffea arabica*^62^ and *Theobroma cacao*^63^ (see Figure S6 for maps with the data points). Because herbarium data can be problematic, we not only discarded duplicates but also the records that fell outside the known altitudinal distribution of coffee and cacao in Latin America and the Caribbean, namely below 350 m and above 2000 m for coffee and above 1300 m for cacao. We also kept records with dates starting in 1980 or later that had at least three decimal places of precision in the long/lat information. We present our models as a working hypothesis of agroforestry areas for plants with ecology similar to *T. cacao* and *C. arabica*, which covers the most common forms of agroforestry in lowland and middle-elevation areas in Latin America and the Caribbean. The final datasets derived from GBIF are provided in Annex 1.

We did not include presence points for *Coffea canephora* because the usable records in GBIF are not only scarce (fewer than 400) but also seem to be mixed with *C. arabica* in some unusual places. Although both species may be planted in close proximity in some ecotones, there is always a risk of misidentification. Therefore, we took a conservative approach and did not attempt an SDM for *C. canephora*. Overall, 2378 points were used for modeling the distribution of coffee and 3725 for cacao. We did not use any method to avoid sampling bias in the data, as crop records are different from wild species data; in fact, crop data are inherently aggregated for socioeconomic reasons, and it is this spatial aggregation that needs to be modeled.

For our simulations, we adapted the MaxEnt approach^64,65^. MaxEnt is a machine learning method used to predict the potential geographic distribution of a species based on its known occurrence and environmental factors. It identifies the probability distribution that is the most uniform (i.e., the one that maximizes entropy) and that aligns with the observed species occurrences. In essence, it attempts to predict where a species is likely to be found based on the conditions that have already been identified as conducive to its survival.

Instead of using the WorldClim climate database used in other coffee and cocoa studies, we chose to use the six TerraClimate variables on which the subsequent correlation analysis was based. See the next section for details on the variables. Initial test runs with the Bioclimatic variables from the WorldClim database showed convergence for zones in the mountainous areas, but large discrepancies in the Amazonian lowlands (results not shown). Therefore, for the sake of consistency in all our analyses, we decided to use only the TerraClimate variables for the SDM. Finally, to increase the realism of our simulations, we added a standardized soil layer^66^ and a biome layer, modified from Dinerstein et al. (2017)^67^, in which the biomes were classified by elevation ranges (0–500 m, 500–1200 m, 1200–1800 m, 1800–2000 m, 2000–3000 m and > 3000 m) using a 90 m digital elevation data from the Shuttle Radar Topography Mission (SRTM 90 m)^68^.

To verify the accuracy of the model, we used high-resolution occurrence data from southeastern Brazil since a comprehensive coffee and cocoa distribution database is not available for the entire Latin America and the Caribbean. For *T. cacao*, we used agroforestry cacao data from Bahía, provided by the MapBiomas Cacau project^69^. For coffee, we used the layer compiled by Gomes et al. (2020)^46^. In both cases, presence data was converted to pixels of the same dimensions as the output of the distribution model, and a proportion of true positives and false negatives was recorded for each crop.

### ENSO and terrestrial climate data

#### ENSO data

We used the Oceanic Niño Index (ONI) Index as the primary indicator of climate variability for the Pacific Ocean for our analyses, as this is the most widely used index for monitoring El Niño and La Niña conditions. The index is provided by the National Oceanic and Atmospheric Administration (NOAA) as a 3-month running mean of sea surface temperature (SST) anomalies (differences from a baseline temperature) in the Niño 3.4 region (5° N–5° S, 120°–170° W). The ONI is calculated using centered 30-year base periods that are updated every five years to account for the increasing trends in ocean temperatures (NOAA 2023b). The threshold for defining El Niño/La Niña corresponds to deviations of +/− 0.5 °C from the average SST. The ONI data were downloaded directly from NOAA^70^.

#### Terrestrial meteorological data

We extracted six meteorological variables from the TerraClimate dataset^71^ to serve as climate exposure indicators for potential agroforestry zones. TerraClimate includes a global dataset from 1958 to 2021, providing monthly climatic and hydrological variables with a spatial resolution of approximately 4 km^71^. We selected three primary (maximum and minimum monthly temperatures and total monthly precipitation) and three derived variables relevant to agroforestry systems. Namely, the Palmer Drought Severity Index (PDSI) which accounts for moisture availability through the water balance equation relating precipitation and potential evapotranspiration^72^; soil moisture, which is the amount of water stored in the soil, used to understand the effects of drought on vegetation^73,74^; and vapor pressure deficit (VPD), which measures the difference (deficit) between the moisture content in the air and its saturation point at a given temperature^75–77^.

#### Terrestrial gross primary productivity data

We used the monthly Gross Primary Production dataset from Bi et al.^38^, which provides the data for GPPshade and GPPsun from 1992 to 2020 with a spatial resolution of 0.05º (approximately 4 km). This dataset is derived from an updated two-leaf light use efficiency (TL-LUE) model and classifies GPP according to sunlit and shaded leaves. Bi and colleagues established the base parameters of their dataset based on global vegetation types (He et al.^40^), including deciduous and evergreen broadleaf forests, mixed forests, cropland, grasslands, open shrublands, savannas, and woody savannas. Detailed equations are presented in Bi et al.^38^ and He et al.^40^. In our analysis, GPPshade and GPPsun were considered the proxies of an idealized agroforestry system with a basic structure of a canopy and shade vegetation. The data were transformed into anomalies in the same way as the TerraClimate data, as described below. Next, for coherence between datasets, we calculated a 3-month running average for each pixel for each month in the same manner as the ONI index.

#### Deforestation mask for filtering vegetation-only GPP data

To avoid spurious correlations with pixels containing deforestation or afforestation events during any time of the study period, we used the cumulative loss pixel band (2001–2022) from Hansen et al.^78^ to filter out all potentially compromised pixels from the GPP database. Before applying the filter, the loss pixel band was upscaled to match the ~ 4 km resolution of the gross primary productivity database layers (Figure S8). We preferred this conservative approach, even if the deforestation event was recent, because it still allows us to estimate a correlation coefficient. In this way, all correlation analyses were performed over the same number of years.

### Statistical analyses

Overall, we used the correlation approach to estimate the extent of teleconnections between the main index related to ENSO and meteorological and ecosystem responses for the potential coffee and cacao areas. In the next sections, we will discuss in detail how we addressed teleconnections, exposure, and sensitivity at the continental and species scales.

#### Differential correlations between El Niño/La Niña and terrestrial climate data

We calculated Pearson correlation coefficients (r) between El Niño and La Niña events, separately, and the six terrestrial climate variables mentioned above selected from the TerraClimate database for the years 1992 to 2020. The TerraClimate data are structured as a monthly global sequence of images for each meteorological variable, resulting in 348 maps (12 months × 29 years) for each of the six variables. First, in an iterative process, we masked all layers based on *Coffea* and *Theobroma* SDM and created a unique time series for each pixel. Subsequently, all six meteorological variables were transformed into anomalies by subtracting the mean value for the period 1992–2020. These anomalies were then detrended to reduce spurious linear correlations between time series, and a 3-month running mean was applied. Finally, we used the ONI thresholds to filter the climatic data of the variables during El Niño and La Niña events. After calculating the Pearson’s correlation coefficient, the results were mapped, showing the differences between El Niño and La Niña, as well as between the *Coffea* and *Theobroma* zones. Only significant correlations (*p* < 0.05) were included in the maps. Data preprocessing and analyses were performed using Google Earth Engine (Gorelick et al.^79^). The Java^®^ code is available on request from the first author or by downloading from the Zenodo repository^80^.

#### Correlations between ONI and gross primary productivity

We calculated two sets of Pearson correlation coefficients (r) between El Niño and La Niña events, separately, and the GPPsun and GPPshade data for the period 1992 to 2020. As with the TerraClimate dataset, 348 layers were analyzed pixel by pixel to extract time series for the correlation analyses. Anomalies were estimated from the extracted time series using the 29-year means, and a 3-month moving average was calculated on the previously detrended time series. The results of the GPP correlations (*p* < 0.05) were mapped across the entire study area, separately for each variable. Since the GPP dataset was not available on the Google Earth Engine platform, preprocessing and parameter estimation were performed locally on a workstation using Python libraries, including Xarray v0.7.1, Matplotlib v3.8.1, Numpy v1.26.0, pandas v2.1.3, and Osgeo-gdal v3.7.3. Due to the computational requirements, parallel processing was performed on an NVIDIA RTX A5000 graphics card using the CuPy v12.2.0 and CuPy-array v0.1.3 libraries (see González-González et al.^80^).

#### Evaluating vulnerability through exposure and sensitivity maps

Before calculating a summary index for exposure (see below) we examined the independence of the six variables by estimating pairwise correlations of the historical time series data between the six meteorological variables at 40 locations in Latin America and the Caribbean known for coffee and cacao production. We found no clear geographic patterns in the pairwise correlations, suggesting independence of the variables (results not shown).

##### Exposure maps

To calculate an exposure index, a stepwise approach using the Entropy Weight Method (EWM) was implemented^81–83^. Rooted in Shannon’s entropy theory, EWM provides a robust framework for quantifying spatial variability and systematically assigning weights to variables based on their influence. This methodology ensures that variables with higher spatial variability exert a greater impact on the computed indices. Widely used in environmental and agricultural studies, EWM enables the integration of diverse variables into unified metrics, offering insights into the exposure and vulnerability of agroforestry systems to climatic extremes. First, as explained above, only pixels with significant correlations (*p* < 0.05) between the six climatic variables (maximum temperature, minimum temperature, vapor pressure deficit, precipitation, soil moisture, and PDSI) and the ONI index were retained for the EWM-based index. For each variable, the exposure signal was derived by assigning values based on their stress-inducing directionality (e.g., positive or negative correlations). The variables were then normalized by dividing each pixel’s absolute value by the total sum of all pixel values for that variable, ensuring comparability across datasets. For variable $\:{X}_{i}$ at pixel $\:j$, the normalized value $\:{P}_{i,j}$ for a pixel was calculated as:

$$\:{P}_{i,j}=\frac{{X}_{i,j}}{{\sum\:}_{j=1}^{n}{X}_{i,j}}$$

Where $\:n$ is the total number of pixels. This step ensures that the sum of all pixel values for a variable equals 1. Next, the entropy $\:{E}_{i}$ of each variable was calculated using the formula:

$$\:{E}_{i}=-k\sum\limits_{j=1}^{n}{P}_{i,j}\times\:ln\left({P}_{i,j}\right)$$

where $\:k=\frac{1}{ln\left(n\right)}$ is a scaling constant ensuring $\:{E}_{i}$ lies between 0 and 1. Higher entropy indicates greater uniformity across pixels, while lower entropy suggests higher spatial variability. The degree of diversification $\:{d}_{i}$ was derived as:

$$\:{d}_{i}=1-{E}_{i}$$

Variables with greater spatial variability (lower entropy) received higher diversification values. Next, weights $\:{w}_{i}$ for each variable were calculated by normalizing the degrees of diversification:

$$\:{w}_{i}=\frac{{d}_{i}}{{\sum\:}_{i=1}^{m}{d}_{i}}$$

where $\:m$ is the number of variables. Finally, a climatic exposure index for each pixel was computed as:

$$\:{exp}_{j}=\sum\limits_{i=1}^{m}{w}_{i\:}\cdot\:\:{X}_{i,j}$$

Separate indices were calculated for El Niño and La Niña phases to ensure that the spatial distributions of phase-specific impacts were captured.

##### Sensitivity maps

Binary sensitivity maps based on GPP correlations with the ONI indices were calculated to examine the overall behavior of the correlations. For GPP, positive correlations between El Niño and GPP for both sun and shade indicate increased productivity with increasing Pacific temperatures during the study period, while positive correlations for La Niña indicate decreased productivity. Conversely, negative correlations indicate decreased productivity for canopy and subcanopy vegetation during El Niño and increased productivity during La Niña. For each GPP map, all pixels with significant Pearson correlations were converted to binary values: +1 for increased productivity and − 1 for decreased productivity, based on the sign of the correlation for El Niño and La Niña. Using this binary classification, a 2 × 2 sensitivity typology was constructed, identifying: (a) zones where both sun and shade vegetation show increased productivity with warmer temperatures; (b) zones where both show decreased productivity with warmer temperatures; and (c) zones where sun and shade vegetation show opposite responses.

#### Timing responses of GPP to ENSO variability

Building on the correlation analysis described in section “Correlations between ONI and gross primary productivity”, we next examined how the strength and spatial distribution of the ONI-GPP relationship evolved through time. To assess the timing and spatial extent of productivity response to ENSO, we computed additional pixel-wise Pearson correlation coefficients between monthly ONI and GPP anomalies for lag intervals from − 3 to + 3 months relative to the ONI peak. We then constructed boxplots for each crop and canopy type (sun and shade) for the seven time lags in months: − 3, − 2, − 1, 0, + 1, +2, + 3. Next, we looked for transition periods for both potential coffee and cacao-growing regions where the median changed between consecutive lag months from positive to negative or viceversa. Once the time lags with median changes were identified, pixels were classified into two categories as changing from non-significant correlations to significant correlations (positive or negative) and a single map was produced. All the analyses were carried out in Numpy Python^80^.

## Supplementary Information

Below is the link to the electronic supplementary material.

Supplementary Material 1

Supplementary Material 2

## Data Availability

The code used to perform the analyses in this study is publicly available via the Zenodo repository at https://doi.org/10.5281/zenodo.14780206.
