# Supplementary material for: Gross primary productivity analyses suggest higher ENSO-mediated impacts in lowland cacao areas compared to mountain coffee regions in Latin America
Source: Sci Rep. 2025 Nov 7;15:39136. doi: 10.1038/s41598-025-27292-3 (PMC12595053; doi:10.1038/s41598-025-27292-3)
Supplement: Supplementary file 1 — Supplementary Material 1 [file 41598_2025_27292_MOESM1_ESM.docx]

**Supplementary Information**

**Gross Primary Productivity analyses suggest higher ENSO-mediated impacts in lowland cacao areas compared to mountain coffee regions in Latin America**

**Andres González-González^*^, Benjamin Quesada, Nicola Clerici and Juan Fernández-Manjarrés**

^*^ Corresponding author: andres.gonzalez@universite-paris-saclay.fr


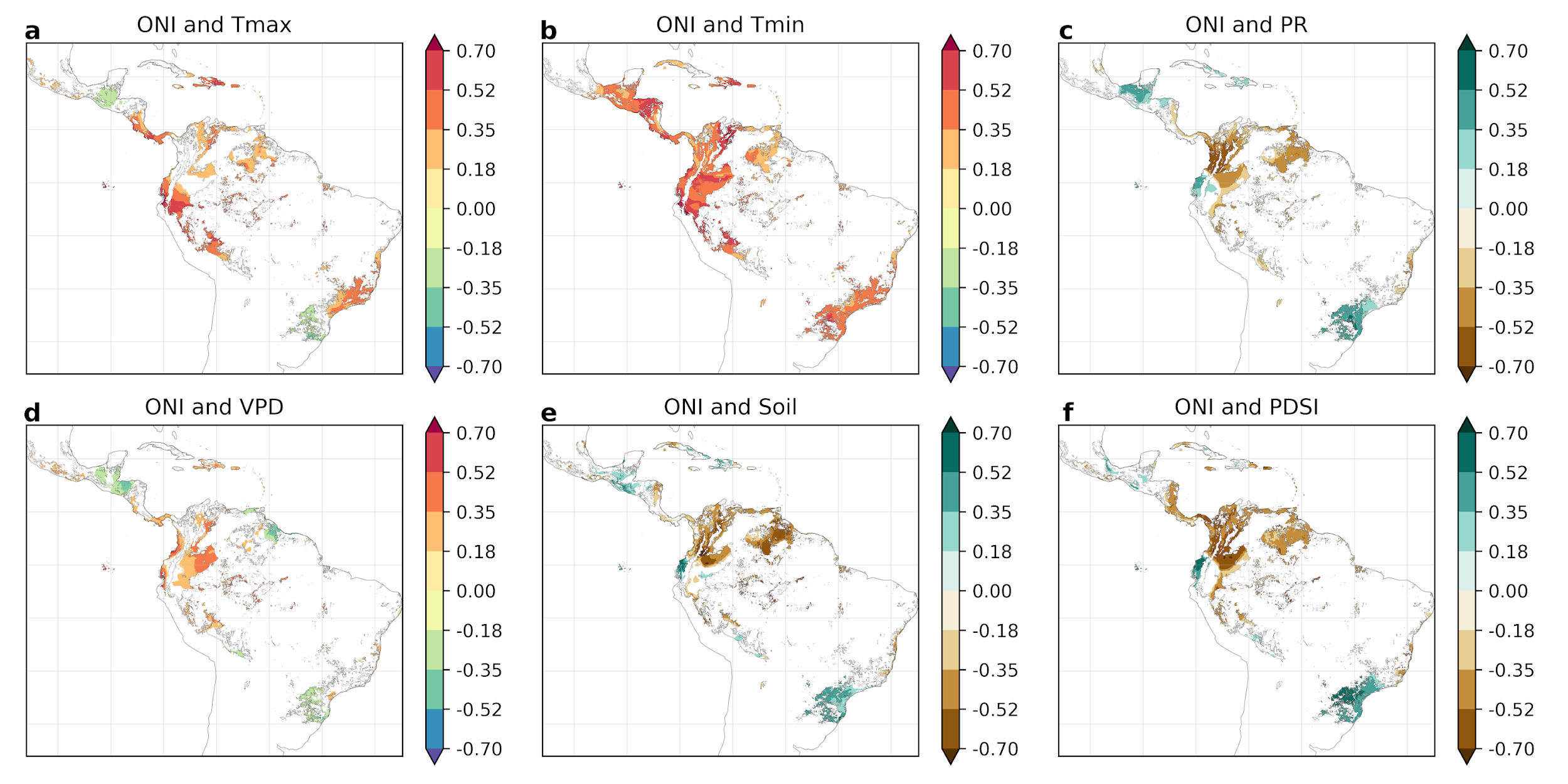


**Figure S1:** Pearson correlation coefficients from 1992 to 2020 between ONI (Oceanic Niño Index) and terrestrial meteorological variables from the TerraClimate dataset during El Niño events (ONI ≥ 0.5°C). Only significant correlations with p-values < 0.05 are shown in the combined *Coffea arabica* and *Theobroma cacao* potential zones. The analyzed variables were Tmax (maximum temperature) (**a**), Tmin (minimum temperature) (**b**), PR (precipitation) (**c**), VPD (vapor pressure deficit) (**d**), Soil (soil moisture) (**e**), and PDSI (Palmer Drought Severity Index) (**f**). Maps generated in Python v3.11 using Matplotlib v3.10.3 (<https://matplotlib.org>) and Cartopy v0.24.1 (<https://scitools.org.uk/cartopy>).


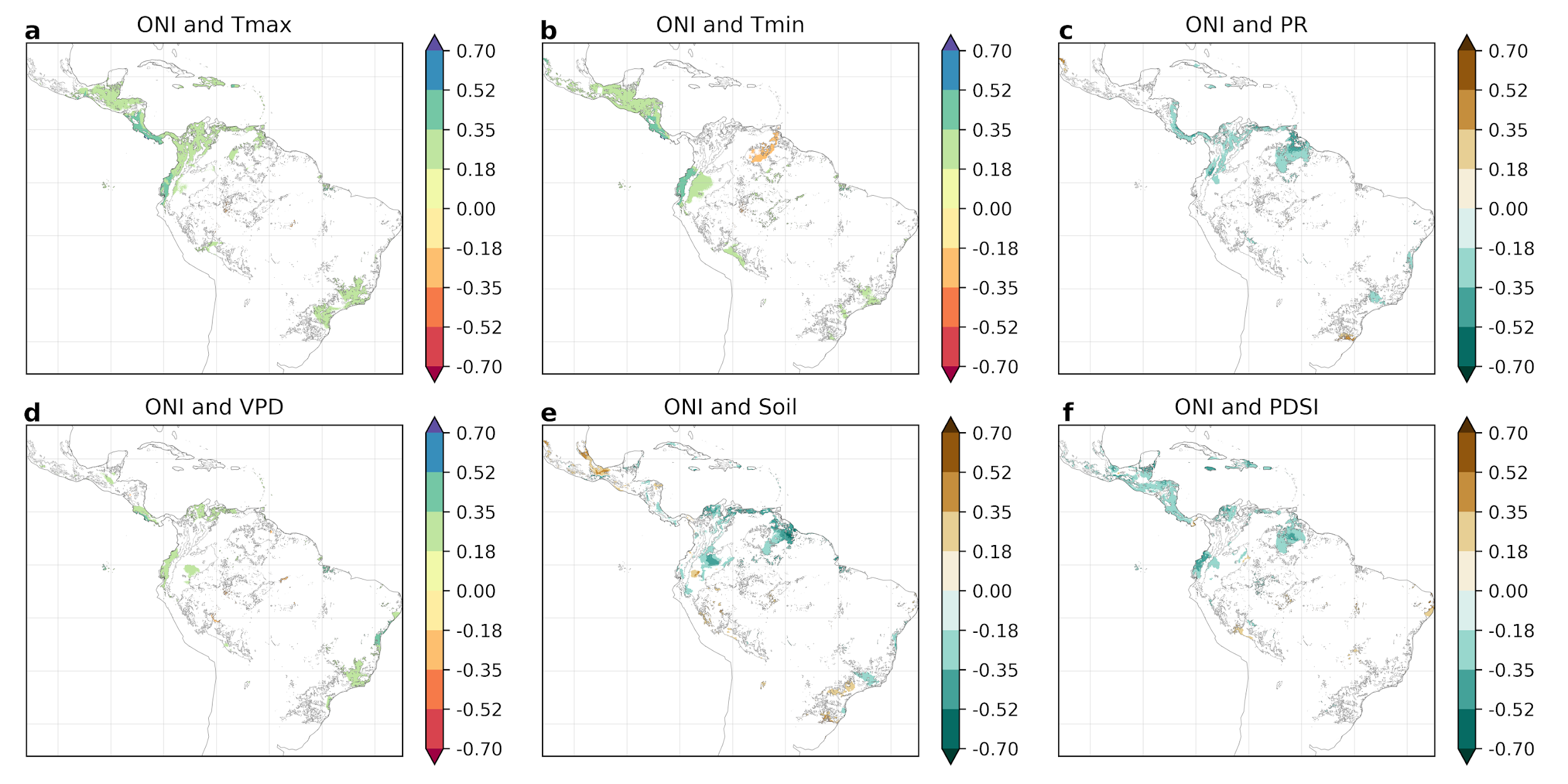


**Figure S2:** Pearson correlation coefficients from 1992 to 2020 between ONI (Oceanic Niño Index) and terrestrial meteorological variables from the TerraClimate 1 dataset during La Niña events (ONI ≤ -0.5). Only significant correlations with p-values < 0.05 are shown in the combined *Coffea arabica* and *Theobroma cacao* zones. The variables analyzed were Tmax (maximum temperature) (**a**), Tmin (minimum temperature) (**b**), PR (precipitation) (**c**), VPD (vapor pressure deficit) (**d**), Soil (soil moisture) (**e**), and PDSI (Palmer Drought Severity Index) (**f**). Maps generated in Python v3.11 using Matplotlib v3.10.3 (<https://matplotlib.org>) and Cartopy v0.24.1 (<https://scitools.org.uk/cartopy>).


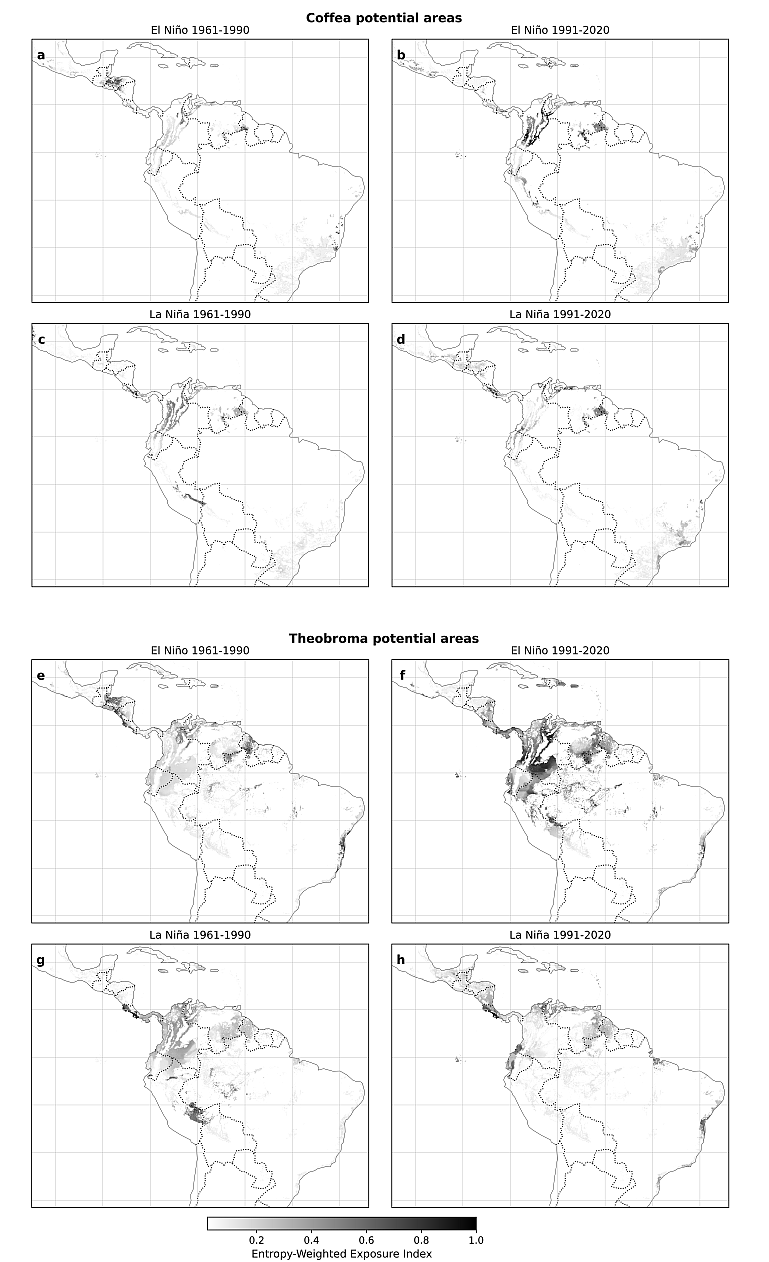


**Figure S3:** Comparison of climate exposure related to the El Niño-Southern Oscillation between the Periods 1961-1990 and 1991-2020 for potential Coffea (a-d) and Theobroma Cacao (e-h) growing areas. Exposure indices were derived from the Pearson correlation coefficients between the Oceanic Niño Index (ONI) and selected TerraClimate variables (see Figures S1 and S2). The indices were quantified using the entropy weighted method (EWM), as described in the online methods section of the main manuscript. Maps generated in Python v3.11 using Matplotlib v3.10.3 (<https://matplotlib.org>) and Cartopy v0.24.1 (<https://scitools.org.uk/cartopy>).


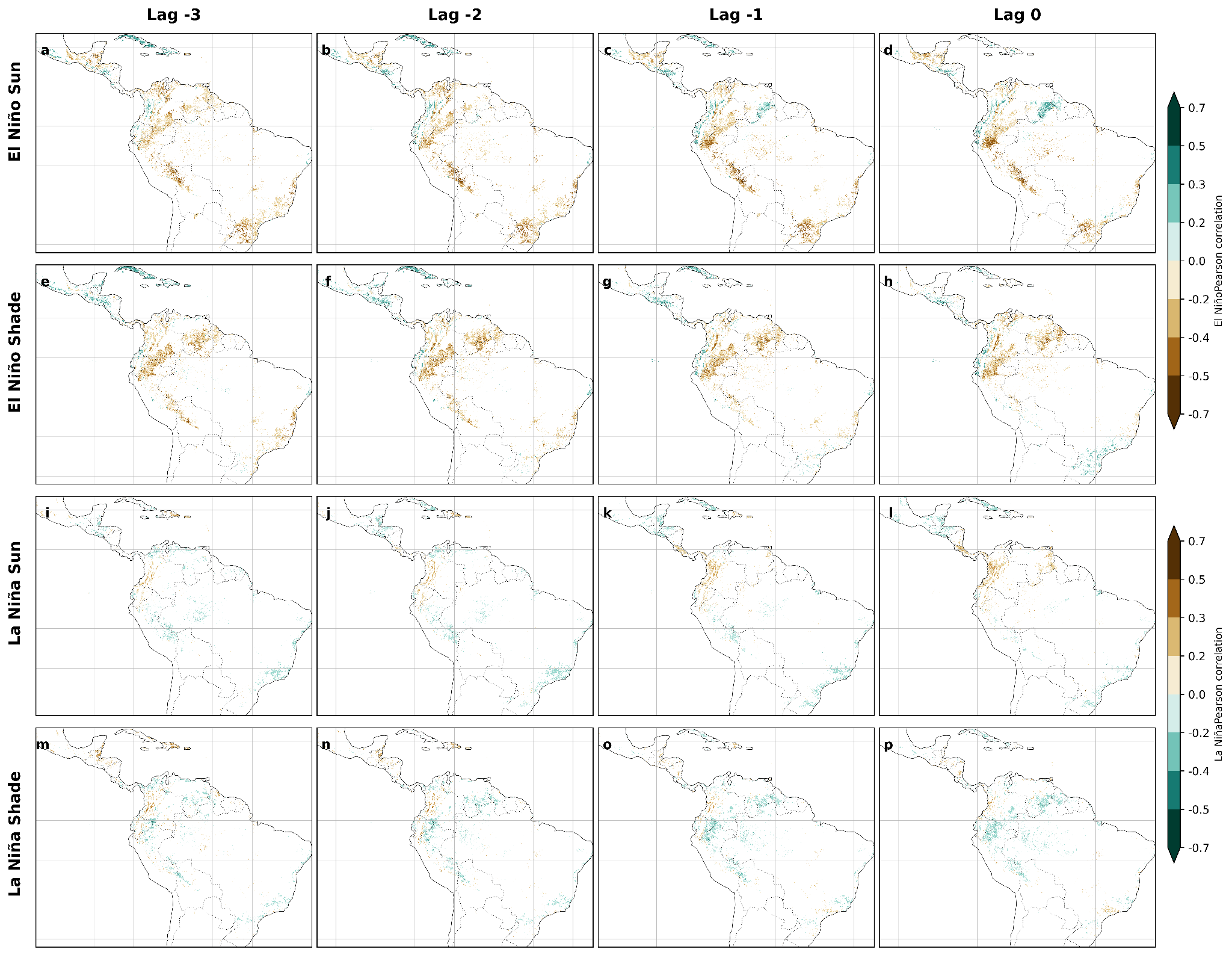


**Figure S4:** Pearson correlation coefficients from 1992 to 2020 between the Oceanic Niño Index (ONI) and Gross Primary Productivity (GPP) metrics from the Bi et al. (2022) dataset at time lags -3, -2, -1 and 0 during El Niño and La Niña events. Both *Coffea arabica* and *Theobroma cacao* potential zones are combined for visual purposes. The first and second rows show the correlations (p > 0.5) between ONI and GPP Sun and GPP Shade, respectively, under El Niño conditions (ONI ≥ 0.5). Likewise, the third and fourth rows show the correlations (p > 0.5) under La Niña conditions (ONI ≤ -0.5). Note that correlations at lag = 0 were used for calculating the sensitivity of GPP in Figure 3 of the main text. Maps generated in Python v3.11 using Matplotlib v3.10.3 (<https://matplotlib.org>) and Cartopy v0.24.1 (<https://scitools.org.uk/cartopy>).


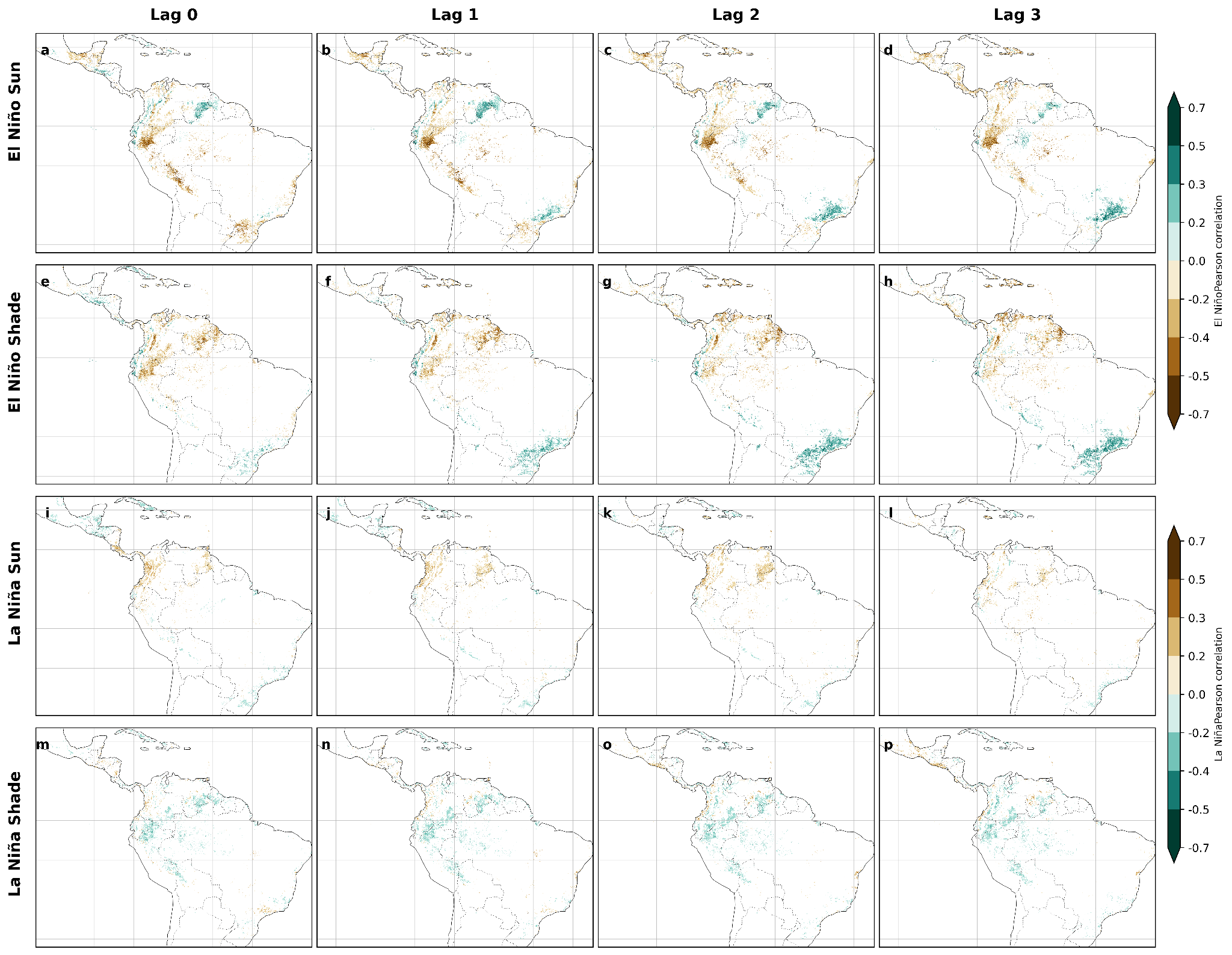


**Figure S5:** Pearson correlation coefficients from 1992 to 2020 between the Oceanic Niño Index (ONI) and Gross Primary Productivity (GPP) metrics from the Bi et al. (2022) dataset at time lags 0, +1, +2 and +3 during El Niño and La Niña events. Both *Coffea arabica* and *Theobroma cacao* potential zones are combined for visual purposes. The first and second rows show the correlations (p > 0.5) between ONI and GPP Sun and GPP Shade, respectively, under El Niño conditions (ONI ≥ 0.5). Likewise, the third and fourth rows show the correlations (p > 0.5) under La Niña conditions (ONI ≤ -0.5). Note that correlations at lag = 0 were used for calculating the sensitivity of GPP in Figure 3 of the main text. Maps generated in Python v3.11 using Matplotlib v3.10.3 (<https://matplotlib.org>) and Cartopy v0.24.1 (<https://scitools.org.uk/cartopy>).


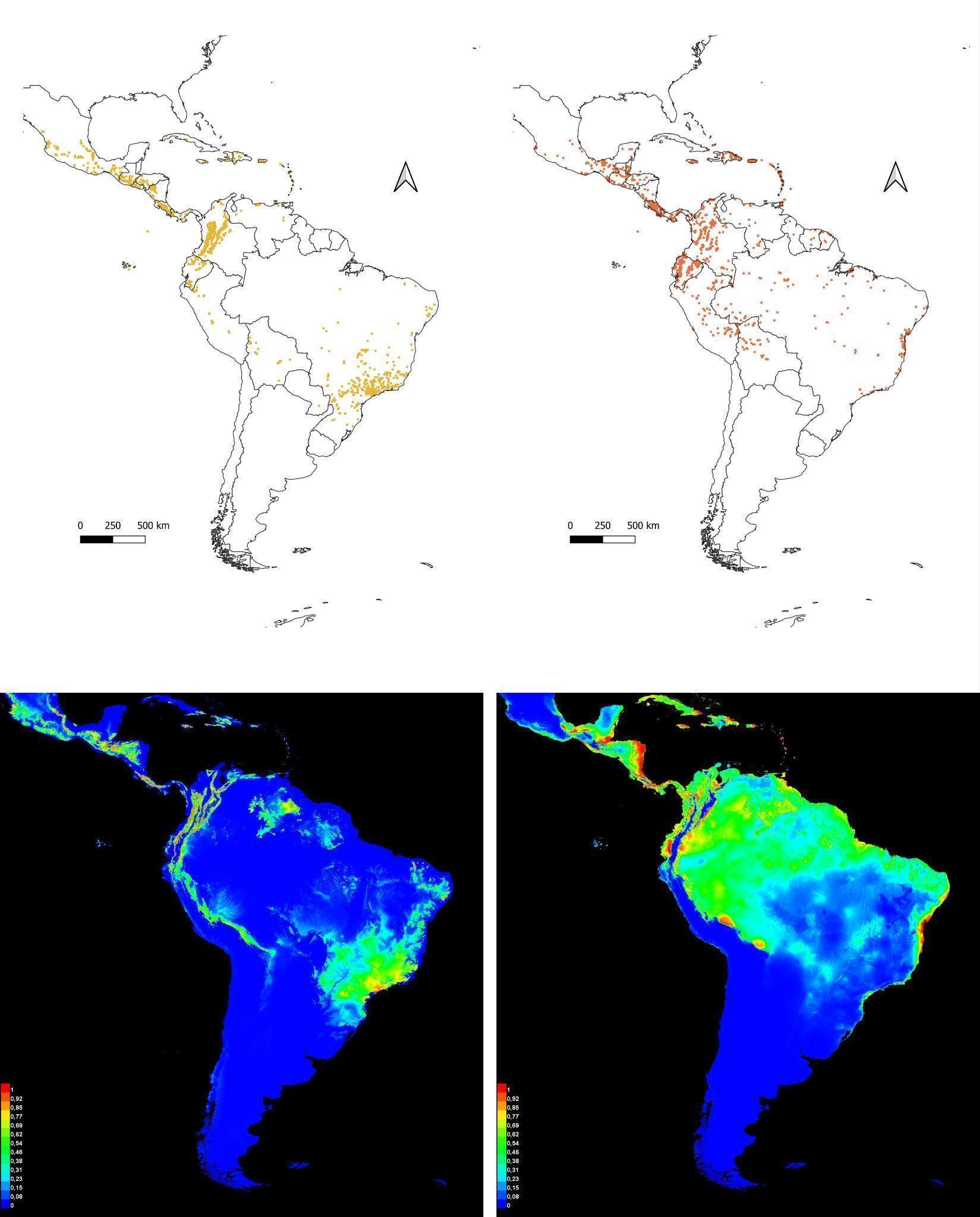


**Figure S6: Upper panel:** field observations from the GBIF database for *Coffea arabica* and *Theobroma cacao*. **Lower panel:** Raw cloglog output from the Maxent Species Distribution Model (SDM). Please see methods for record selection and modelling parameters. The upper panel maps were generated with QGIS v3.34.2-Prizren (QGIS Development Team, 2024; <https://qgis.org>) and the lower panel maps correspond to figures generated by MaxENT v3.4.3 (American Museum of Natural History, 2024; https://biodiversityinformatics.amnh.org/open_source/maxent/).


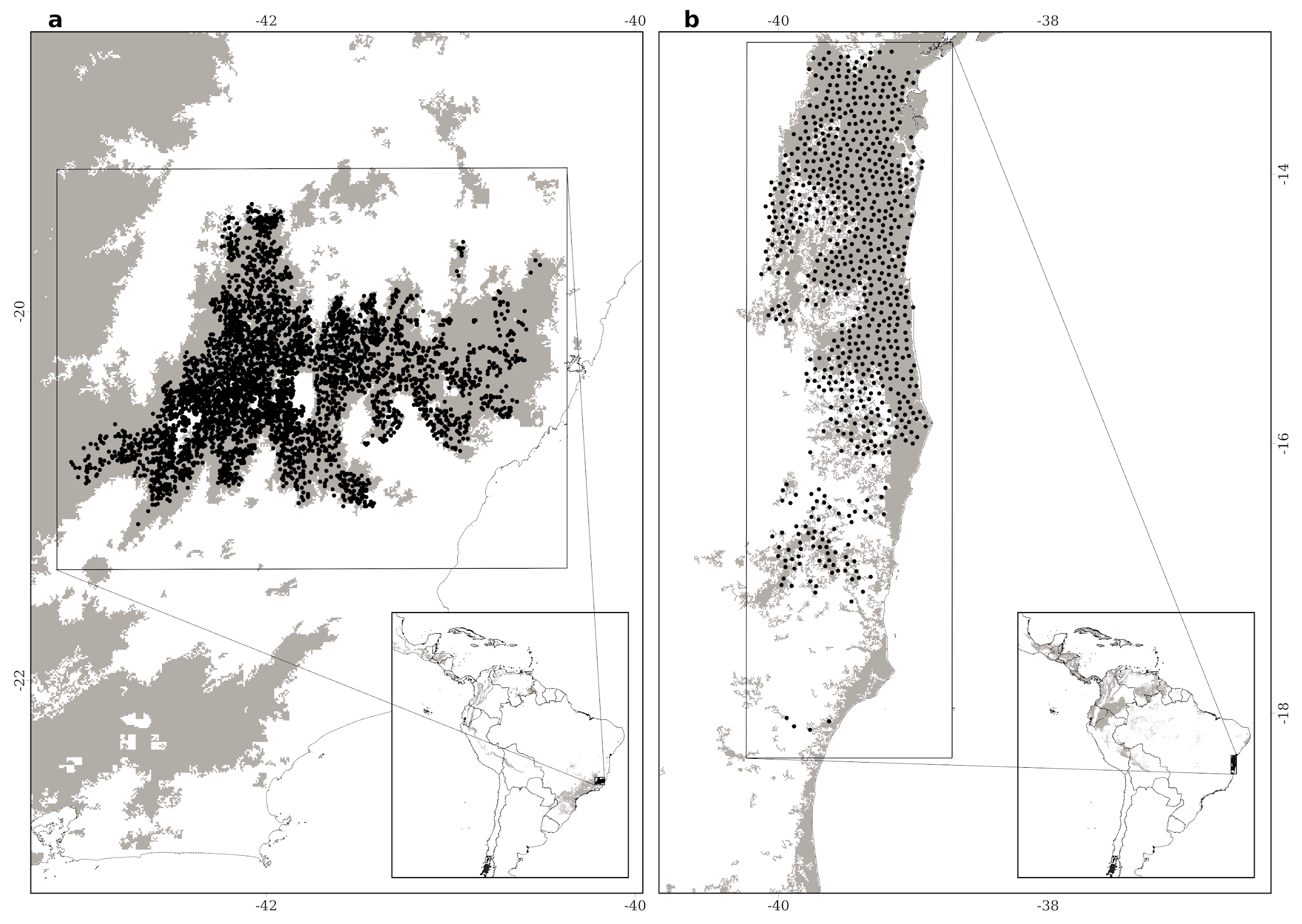


**Figure S7. Spatial validation points for recall calculation in coffee and cacao suitability analysis.** Panel **a.** corresponds to the geographic distribution of *Coffea arabica* validation points (black dots) across suitability areas (shaded in gray). The points obtained from Gomes et al. (2020) were used to calculate recall, yielding 87% true positives and 13% false positives. Panel **b.** shows the distribution of *Theobroma cacao* validation points (black dots) within modeled suitability areas in Bahia, Brazil, with data obtained from MapBiomas Cacau. For cacao, the recall analysis yielded 79% true positives and 21% false positives. Map created using QGIS v3.34.2-Prizren (QGIS Development Team, 2024; https://qgis.org).


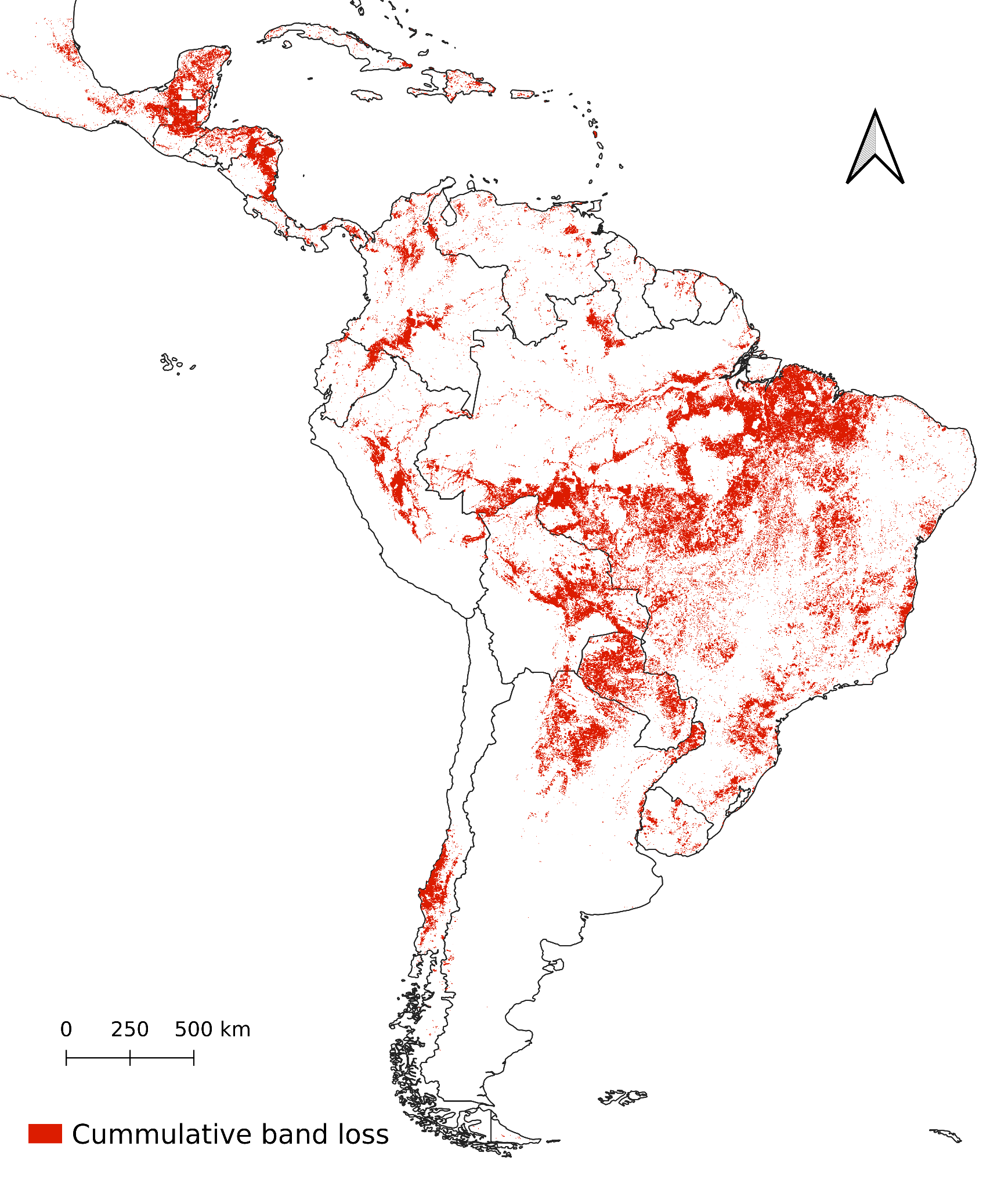


**Figure S8:** Cumulative Forest Cover Loss (2001-2022) Based on the Hansen et al. (2013) Dataset (Global Forest Change v1.11). This map was used as a mask to filter pixels from the Bi et al. (2022) database. Map created using QGIS v3.34.2-Prizren (QGIS Development Team, 2024; https://qgis.org).
